# Snapshot of Obstetric National Audit and Research Project (SONAR1): aprotocol for an international observational cohort study

**DOI:** 10.1136/bmjopen-2025-103525

**Published:** 2025-06-24

**Authors:** Reshma Patel, James O’Carroll, Justin Kua, Pervez Sultan, Brendan Carvalho, Nadir El Sharawi, Victoria Eley, Pat O’Brien, Susanna Stanford, Samantha Hill, Robert Craig, Nuala Lucas, Bo Hou, S Ramani Moonesinghe

**Affiliations:** 1 Theatres and Anaesthetics, University College London Hospitals NHS Foundation Trust, London, UK; 2 Department of Surgery and Targeted Intervention, University College London, London, UK; 3 Central London Patient Safety Research Collaboration, NIHR, London, UK; 4 Health Services Research Centre, National Institute For Academic Anaesthesia, London, UK; 5 Department of Anaesthesia, St Paul’s Hospital, Vancouver, British Columbia, Canada; 6 Stanford University, Stanford, California, USA; 7 University of Arkansas for Medical Sciences Department of Anesthesiology, Little Rock, Arkansas, USA; 8 Royal Brisbane and Women’s Hospital, Herston, Queensland, Australia; 9 Maternal Fetal Medicine, Institute for Women’s Health, University College London Hospitals NHS Foundation Trust, London, UK; 10 Patient Representative, Northumbria, UK; 11 Patient Representative, Sussex, UK; 12 Northwick Park Hospital, Harrow, UK; 13 Centre for Perioperative Medicine, Research Department of Targeted Intervention, University College London, London, UK; 14 Health Services Research Centre, Royal College of Anaesthetists, London, UK

**Keywords:** Cesarean Section, Anaesthesia in obstetrics, Adult anaesthesia, Pain management, Clinical Protocols, Clinical Trial

## Abstract

**Introduction:**

Caesarean birth (CB) under neuraxial anaesthesia (NA) is the most performed inpatient operation in the UK. The incidence of intraoperative pain during caesarean delivery performed under neuraxial anaesthesia is unclear, with limited data that used patient-reported measures to investigate intraoperative pain. The short- and medium-term impacts on patients of this adverse event are unknown.

**Methods and analysis:**

We will undertake a multicentre, prospective observational cohort study to evaluate the incidence and impact of pain experienced by patients during CB performed under neuraxial anaesthesia. Routine audit data will be collected for all patients undergoing caesarean delivery for any indication during a 1 week window at participating hospitals within the UK and Queensland, Australia. The dataset will include patient, anaesthetic, obstetric and neonatal risk factors for intraoperative pain. Local investigators will then seek informed consent from patients either before or within 24 hours of delivery to record patient experience and patient-reported outcomes at 24 hours and 6 weeks postdelivery. Local investigators at participating hospitals will also complete a survey evaluating compliance with evidence-based structural standards at their sites. The patient characteristics, structures, processes and outcomes will be described. Inferential techniques will be used to evaluate the relationship between risk factors and postoperative outcomes.

**Ethics and dissemination:**

This study received ethical approval from the Leicester Health Research Authority and Care Research Wales, REC reference 24/EM/0084) on 24 May 24. The study received ethical approval from the Human Research Ethics Committee of Metro North Health in Australia on 25 March 2024 (REC Ref HREC/2024/MNHA/103767). The results of the study will be reported in accordance with the Strengthening the Reporting of Observational Studies in Epidemiology statement. The results will be disseminated via conference presentations, peer-reviewed academic journals and reports prepared for patients, the public and policy makers.

**Trial registration number:**

ISRCTN15269213.

**STRENGTHS AND LIMITATIONS OF THIS STUDY**

- This comprehensive study aims to recruit patients from across the UK and Australia.
- This study uses patient-centric outcome measures and patient-reported data for its primary and secondary outcomes.
- This study focuses on an important patient concern and aims to investigate how this may impact 6-week postpartum recovery.
- The snapshot nature of the study means that system pressures identified may not be generalisable.
- Those patients most affected by the primary outcome may choose not to participate in the study.

## Introduction

 Caesarean birth (CB) is the most performed inpatient surgical procedure worldwide.^1^ The CB rate is rising in most countries. In the UK, over 136 000 patients between March 2023 and 2024 were delivered by this mode, representing 42% of all deliveries.^2^ Neuraxial anaesthesia techniques are associated with lower neonatal and maternal morbidity^3^ and are considered to be the gold standard anaesthesia for CB.^4^ These techniques are preferable to general anaesthesia as they are associated with lower rates of neonatal and maternal morbidity, while promoting improved maternal–infant bonding, earlier breastfeeding, partner presence during surgery and superior postpartum recovery.^5^ However, the effectiveness of neuraxial anaesthesia is not absolute, with failure rates and intraoperative pain incidence varying widely due to a complex interplay of patient-specific factors, clinician experience, anaesthetic technique, obstetric conditions, neonatal conditions and institutional protocols.^6^ Furthermore, there is a lack of consistency in the literature on how intraoperative pain during CB should be measured and managed, and for this reason, the true incidence of intraoperative pain remains unclear. Published data on the incidence of pain during CB are predominantly from single centre studies, with incidence rates ranging widely from 1.7%^7^ to 33%.^8^,^10^ A systematic review of randomised controlled trials in patients having a scheduled CB reported the overall incidence of pain, defined as requirement for unplanned supplemental analgesia, repeat neuraxial technique or conversion to general anaesthesia, as 14.6%.^11^

Current guidance emphasises the risk of adverse psychological sequelae as a result of pain during CB and provides expert recommendations on its management; however, the scale of the potential impact of intraoperative pain experienced on short- and medium-term on the patient remains insufficiently explored.^6^ There have been no prospective studies evaluating neuraxial anaesthesia from the patient’s perspective, how intraoperative pain is managed, and what the outcomes are for those that experience this adverse event.

Few studies in the past decade have evaluated pain during CB using contemporary obstetric anaesthesia practices and techniques. During this time, there have been changes to both anaesthetic and obstetric staffing levels within obstetric units in the UK. Advances in labour ward epidural analgesia include the use of changes to practices in ‘topping-up’ epidurals, increased use of lower concentration epidural local anaesthetic for labour analgesia, addition of techniques into clinical practice to initiate and maintain labour analgesia and surgical anaesthesia, including combined spinal epidural or dural puncture epidural and developments in labour analgesia delivery strategies and epidural pump technology. This is particularly relevant as labour epidural analgesia is frequently converted to surgical anaesthesia for intrapartum CB.

Therefore, we plan to conduct an international prospective, observational cohort study to address this knowledge gap. The aims of the study are to elucidate the incidence, risk factors and sequelae of pain experienced during CB performed under neuraxial anaesthesia; and to use patient-reported outcome measures (PROMs) to evaluate the physical and psychological impact on those who experience intraoperative pain.

## Methods

### Key study dates

The study opened for data collection to national sites on 17 March 2025 and closed to new patient recruitment on 17 May 2025. We expect all data collection to be complete by 1 August 2025.

### Study design

Snapshot Obstetric National Anaesthetic Research Project (SONAR1) is a prospective, multicentre, observational cohort study. The study has two components:

1. Patient study: cohort study of patients undergoing CB under neuraxial anaesthesia in participating hospitals
2. Organisational survey: study of institutional and structural risk factors that may influence incidence and/or outcomes for patients during CB.

A pilot study has been completed at University College London Hospital, enabling us to refine and modify the study protocol and the final version to be delivered across the UK and Australia.

### Research questions

RQ1: What is the incidence of intraoperative pain reported by patients during CB performed with neuraxial anaesthesia?

RQ2 What is the incidence of incidence of intraoperative pain reported by clinicians during CB performed with neuraxial anaesthesia?

RQ3: What are the hospital-, patient-, clinician-, anaesthetic- and obstetric-related factors associated with the incidence of pain during CB performed under neuraxial anaesthesia?

RQ4: What are the hospital-, clinician-, patient-, anaesthetic- and obstetric-related factors associated with the short- and longer-term outcomes following CB performed under neuraxial anaesthesia?

### Objectives

To estimate the incidence of patient and clinician-reported intraoperative pain during CB conducted with neuraxial anaesthesia. (RQ1)

To describe how intraoperative pain is managed, and to describe how successful practice strategies are. (RQ2,3)

To evaluate day 1 and 6-week patient reported outcomes related to intraoperative pain during CB (RQ1,2,3)

To evaluate the physical and psychological impact on patients of CB in the short- and medium-term using PROMs. (RQ 2,3)

## Eligibility criteria

### Hospital level

All hospitals within the United Kingdom’s National Health Service (NHS) and Australian public hospitals with a consultant-led obstetric unit and anaesthetic support are eligible to take part.

On completion of a site initiation visit, local principal investigators (PI) will be provided with access to an online organisational survey. They will be asked to complete this in conjunction with the institutions’ clinical lead for obstetric anaesthesia. The survey explores the preoperative, intraoperative, postoperative and structural factors that may influence the incidence or impact of intraoperative pain during CB. The information will be used to examine hospital and structural risk factors that may impact the incidence, management and follow-up of intraoperative pain.^6 12^

### Patient level

#### Inclusion criteria

1. Patients who are 18 years or over
2. Undergoing a CB that is scheduled or unscheduled of any urgency category
3. ≥32 weeks’ gestation
4. Neuraxial anaesthesia is the planned primary mode of anaesthesia.

#### Exclusion criteria

1. Patient refusal
2. Other modes of birth or obstetric surgical procedures, such as instrumental delivery, are not eligible
3. CB where a general anaesthetic is planned as the primary mode of anaesthesia
4. Neonatal or fetal demise

### Consent

As early as possible following admission to a participating maternity unit, all potential participants who meet the inclusion criteria will, wherever possible, be provided with information about the study via a participant information sheet (PIS). Any member of the clinical or research staff can give the participant a patient informational sheet and inform them that the study is occurring within the facility, but any specific participant questions regarding the study should be directed to a local investigator. In most cases, provision of information regarding SONAR1 will be provided shortly following admission and before delivery. However, in emergencies or for other local logistical reasons, some participants may only receive the patient information sheet following delivery. In this case, local investigators are required to ensure that the participant is given at least 1 hour to consider the information on the patient information sheet before being approached to consent.

**Figure 1:**
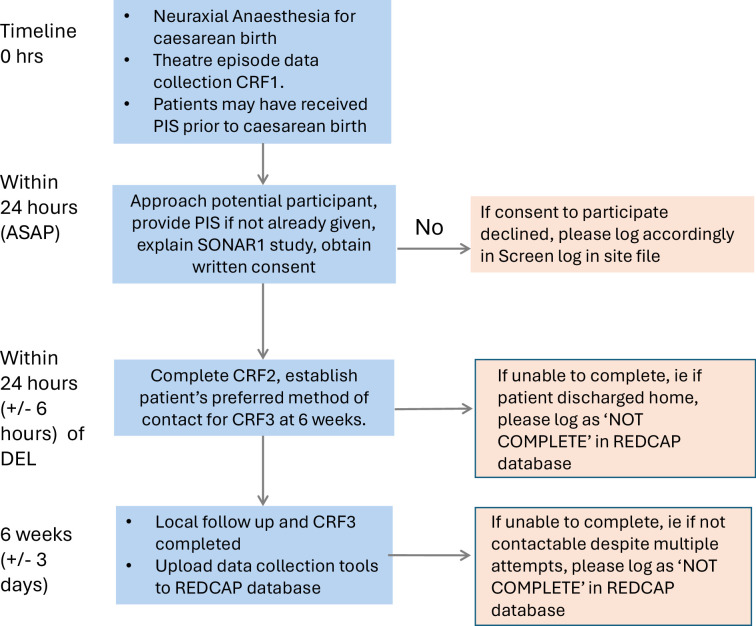
Flow chart for eligible patient. CRF, case record form; PIS, participant information sheet; REDCap, Research Electronic Data Capture; SONAR1, Snapshot Obstetric National Anaesthetic Research Project; ASAP, as soon as possible; DEL, delivery.

Patients who meet the inclusion criteria will be approached to provide informed consent to participate at the earliest feasible opportunity postoperatively during inpatient hospitalisation. Consent can be sought by anyone who has completed the required training to obtain consent according to local guidelines—this may include; clinical staff, research nurses, research midwives or non-clinical research assistants. If participants are too unwell to be approached during the immediate postoperative period, local investigators should use their judgement about the appropriate timing to approach for consent. For patients who are admitted to high dependency or intensive care postpartum, local investigators should use their clinical judgement as to whether a patient can be approached to participate. This process is summarised by figure 1, demonstrating the planned flow chart for each eligible patient.

## Data collection and follow-up procedures: summary

Data will be collected by local investigators using three case record forms (CRFs). CRF1 does not require patient consent and consists of routine patient, obstetric and anaesthetic audit data that are collected during the CB. It is completed by the attending anaesthetist present for the CB for all patients. Patients who provide informed consent to participation in the study will be approached to complete CRF 2 within 24 (±6 hours) of surgery. Finally, at 6 weeks (±3 days) following delivery, consenting participants will be contacted by local investigators by telephone, to complete CRF3.

### Case record forms

All case record forms discussed here are available in online supplemental file 1.

Perioperative anaesthetists will complete CRF 1 for every patient during the study period. If perioperative anaesthetists are unable to fully complete the CRF1 at the time of surgery, local investigators can complete the CRF1 retrospectively, through accessing patient records.

Eligible patients will be asked to complete CRF2 with local study investigators within 24 (±6) hours of their delivery. CRF2 includes a previously described and validated PROM: the Maternal Satisfaction Score^13^ and additional questions to evaluate intraoperative experience and satisfaction with anaesthesia and analgesia during their delivery. Patients will also be asked to provide their contact details to allow local investigators to contact them at 6 weeks (±3 days) for the purpose of completing *CRF3* (detailed below). Patients may have visits from family and other healthcare workers at this time; therefore, it is acceptable to return for more than one visit. Predetermined data collection visit times are encouraged, if within the data collection timeframe and investigator resources allow. The study documents are available in the six most commonly spoken languages in patients who do not speak English, and Welsh. Local investigators should attempt to make it possible, using their local interpretative services to ensure patients who do not speak English are able to take part, if they wish. The patients will need to be able to complete CRF2 with an investigator.

**Figure 2:**
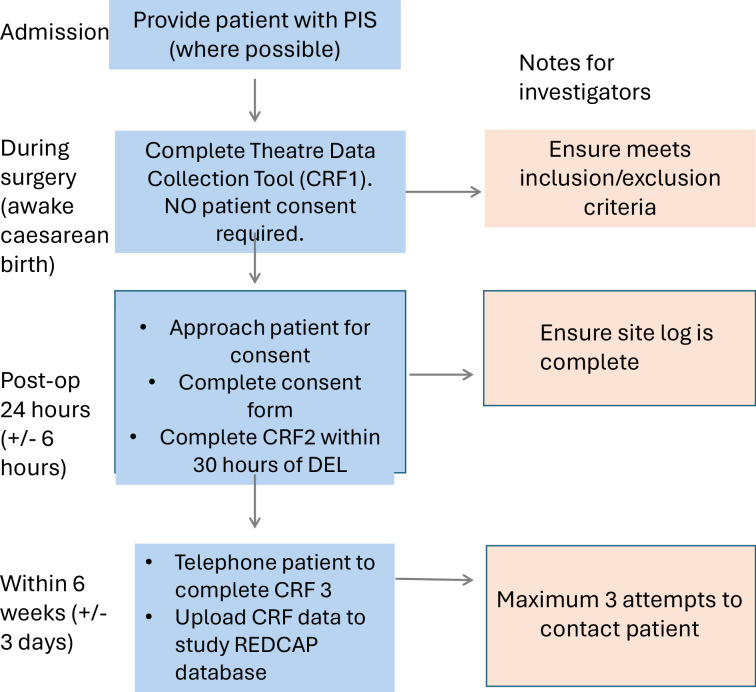
Flow chart of methodology for data collection and follow-up procedures. CRF, case record form; PIS, participant information sheet; REDCap, Research Electronic Data Capture; DEL, delivery.

CRF3—This CRF will be used to collect follow-up data at 6 weeks (±3 days) from participants who provide consent. The PROMs used will be the validated Edinburgh Postnatal Depression Scale (EPDS), the Post-Traumatic Stress Disorder (PTSD) Checklist for *DSM-5* (PCL-5) and Generalised Anxiety Disorder- 7 Scale (GAD7). In addition, the patient will be asked regarding pain at rest and on movement. Patient follow-up will be conducted over the telephone by local investigators. A maximum of three attempts will be made to contact participants, with each attempt being noted on the study log. If no follow-up is completed after three attempts, no further attempts will be made, and the participant should be deemed ‘lost to follow-up’. This should be recorded as such on the study log. If contact is made, but the participant asks to be called back later, it is acceptable to make more than three phone calls. If contact with the participant is made, but *CRF3* is only partially completed (eg, due to other time commitments), it is acceptable to make more than three attempts to recontact; however, recontact should be done with sensible judgement—local investigators should be dissuaded from an excessive number of attempts which could be deemed to be too intrusive by consenting participants. If the patient’s first language is not English and they have consented, they are able to complete CRF3 via telephone using telephone translation services locally. This process is summarised in figure 2, which demonstrates the methodology of data collection for each patient.

In the event of a participating patient scoring >11 for EPDS, 30 for PCL-5 or >10 for GAD-7, the participant should be informed that her score would suggest she has screened positively for postnatal depression, PTSD or anxiety, respectively. The participant should be encouraged to see their general practitioner (GP) or health visitor as soon as possible. If the patient reported suicidal ideation (Q.10 of EPDS), they should be advised to seek immediate medical help, for example, emergency department (ED) visit. Local investigators should offer a letter that details resources available to the patient to support their mental health. If the patient consents, a letter will also be sent to their GP explaining the study and that the patient has been flagged as at risk of either postnatal depression, PTSD or anxiety. If a patient responds with anything other than ‘Never’ for EPDS question 10 regarding self-harm, or if the investigator has concerns regarding their safety or that of the participant’s family, then as well as the letters of resources, the local health visitor teams or local maternity safeguarding teams should be informed. The patient should be advised to visit their GP, contact their health visitors or attend local EDs if appropriate. Local investigators should also contact their local hospital maternity safeguarding team according to local protocols if they have any concerns arising from their interaction with study participants.

### Organisational survey

No follow-up for the organisational survey will be required.

### Outcome measures

Our primary outcome will be the incidence of intraoperative pain as reported by patients in CRF2. Our secondary outcomes will include the incidence of physician-reported pain, incidence of depression, anxiety and/or PTSD from screening at 6 weeks (± 3 days), and pain scores at both 24 hours (±6 hours) and 6 weeks (±3 days). Maternal Comfort and Maternal Satisfaction Scores at 24 hours (±6 hours) will also be secondary outcome measures. A further secondary outcome will be to describe the current practice of how intraoperative pain is managed.

## Analysis plan

### Descriptive statistics

The descriptive epidemiology of the patient, hospital, clinician obstetric and anaesthetic data collected in CRF1 will be reported. Frequency and row percentages will be presented for categorical and binary variables, while median and (IQRs) interquartile ranges will be provided for continuous variables. We will report the incidence of pain during CB (from both patient and clinician perspectives) and how this was managed.

### Inferential statistics

Inferential statistics will be used to understand modifiable and non-modifiable risk factors for pain during CB and self-reported outcomes measured. This analysis will also enable us to explore the relationships between patient and structural risk factors, care processes, short-term and longer-term outcomes. This will include regression modelling to determine factors associated with intraoperative pain, including patient, anaesthetic and obstetric risk factors, and institutional and country level structural and process indicators. Where appropriate, we will use multilevel multivariable regression to investigate these relationships and account for hospital-level clustering.

The results of the study will be reported in accordance with the Strengthening the Reporting of Observational Studies in Epidemiology (STROBE) statement.^14^

### Data sharing

Data will be available to share on reasonable request to the data custodian following relevant local ethical approvals. Each site will be responsible for uploading their data to Research Electronic Data Capture (REDCap).^15^ Each site will only be able to add data for their own patients and will not be able to see or modify data for any other site. The central study team will receive anonymised data only; thus, a data monitoring committee is not required.

### Sample size calculation

This multicentre study will take place over 1 week (each site will collect data for 1 week during a 2-month study period), aiming to recruit at least 1500 participants. The sample size is calculated for detection of patient-experienced intraoperative pain, which is estimated to be 15% based on single-centre pilot study data, with a 95% CI and a margin of error of ±3%. This gives a total of approximately 676 participants.

However, since there will be multiple centres in the study, a design effect has been used to account for between site variation and within site clustering. The design effect is 1+ICC*(n−1) and n is the number of subjects per cluster. As we do not have empirical data for ICC, we have used 2 as the design effect. So, the n is 676*2, giving a total sample size of approximately 1352 participants. We estimate up to 10% will have incomplete data within their CRFs. At 6 weeks, we anticipate approximately 70% of participants will be followed up. This therefore gives a total sample size of 1500 participants with complete baseline, 24 hours and 6-week follow-up data.

### Study management

The day-to-day running of the study will be performed by the study coordinator, in conjunction with the chief investigator and core members. A study management group will meet monthly providing updates on the delivery of the study. Finally, a study oversight board, independent of the study team, will meet to provide independent advice and guidance.

### Patient and public involvement

The themes of this study were prompted by a personal experience of pain during CB.^16^ We have sought public and patient involvement and input from a community-based parent group in North London, comprised people who have given birth at UCLH hospitals within the last 2 years. Two patient/public representatives with relevant experience are members of the study group, playing an equal part in decision-making to clinician and scientist members. The study documents, including the development of the protocol, participant information leaflet, participant information poster, letters for participant and GP, consent form and all CRFs, have been co-designed and ratified with the study team and patient and public representatives.

### Ethics and dissemination

This study received ethical approval from the Leicester Health Research Authority and Care Research Wales, REC reference 24/EM/0084) on 24 May 24. The study received ethical approval from the Human Research Ethics Committee of Metro North Health in Australia on 25 March 2024 (REC Ref HREC/2024/MNHA/103767). The trial is registered with the ISRCTN (ISRCTN15269213, https://doi.org./10.1186/ISRCTN15269213). The results of the study will be reported in accordance with the STROBE statement.

Data for the patient cohort will be first collected on paper CRFs before being transcribed onto electronic CRFs via a dedicated, secure online web tool. Completed paper CRFs were held in a secure location accessible by the local researchers in accordance with Good Clinical Practice guidelines and local information and research governance frameworks.

Data will be entered electronically via a secure, encrypted connection onto an online portal hosted by University College London. The software used for data capture will be REDCap (http://www.project-redcap.org), a secure web application for building and managing online surveys and databases. Access to the web tool is limited to local site Principal Investigators and two other users at local sites that are nominated by local Principal Investigtors. These were password-protected user accounts, and encrypted server connected using Hypertext Transfer Protocol Secure. No local site will have access to any other site information other than their own. An anonymised dataset will be uploaded onto the centralised study database via University College London’s (UCL) REDCAP web-based portal hosted on secure UCL servers. No personal identifiable data from the hospital CRF paperwork will be uploaded to REDCap.

The study is compliant with the requirements of General Data Protection Regulation (2016/679) and the UK Data Protection Act (2018). All investigators and study site staff will comply with the requirements of the General Data Protection Regulation (2016/679) with regards to the collection, storage, processing and disclosure of personal information, and will uphold the Act’s core principles.

The results will be disseminated via conference presentations, peer-reviewed academic journals and reports prepared for patients, the public and policy makers.

### Dissemination and transparency policy

We intend to present the results of SONAR1 in peer-reviewed scientific journals and at medical conferences. Local Principal Investigators and other investigators will be individually named as collaborators for their support. In addition to academic publications, we will provide specific summary reports for the following groups:

1. Partner Colleges—this will include the Royal College of Anaesthetists, Royal College of Obstetricians and Gynaecologists and Australian and New Zealand College of Anaesthetists and Royal Australian and New Zealand College of Obstetricians and Gynaecologists
2. Patients and the Public—our lay representatives will provide support in our dissemination to the non-medical audience.
3. Participating NHS Trusts and Health Boards—all NHS chief executives of all participating organisations will be sent a summary of the key findings.

The study investigators and steering committee have agreed a policy for authorship and contributor status for all manuscripts which arise from this study.

### Conclusions

SONAR1 will use a multi-centred prospective study design and PROMs to obtain detailed information from a comprehensive sample of patients having a CB in the UK and Australia to investigate the incidence of intraoperative pain, neuraxial failure and the impact on short and medium-term outcomes for patients.

## Supplementary material

10.1136/bmjopen-2025-103525 online supplemental file 1

10.1136/bmjopen-2025-103525 online supplemental file 2
