# Supplementary material for: Snapshot of Obstetric National Audit and Research Project (SONAR1): aprotocol for an international observational cohort study
Source: BMJ Open. 2025 Jun 24;15(6):e103525. doi: 10.1136/bmjopen-2025-103525 (PMC12198837; doi:10.1136/bmjopen-2025-103525)
Supplement: online supplemental file 1 [file bmjopen-15-6-s001.pdf]

Snapshot Obstetric National Anaesthetic Research Project – SONAR 1

Case Record Form 1 – Intraoperative data from caesarean delivery

To be completed by the anaesthetist clinically present for the case are requested to complete CRF 1 for all patients other than de novo General Anaesthesia. Please complete as much of the demographic, obstetric, and anaesthetic information at possible. If there are gaps, please ask the patient where possible.

Part 1 Patient demographics

1.1 DAY of caesarean (e.g. Monday)

**1.2.1 Hospital ID: (NOT TRANSFERRED TO RedCAP)** ____________________	**1.2.2 Date dd/mm/yyyy** (NOT TRANSFERRED TO RedCAP)
**1.3 Age**______	**1.4.1 Booking Weight** ______________kg<br>**1.4.2 Height** ______________cm<br>**1.4.3 BMI** ______________kg/m2

1.5 1st /preferred language:
- ☐ English
- ☐ Any other language

1.5.1 If other please (Please state) ___________________

1.6 ASA Grade (noting ASA 2 due to pregnancy)
- ☐ 2
- ☐ 3
- ☐ 4

1.7 Past Medical/Obstetric History (please tick all that apply)

**1.7.1 Cardiovascular**<br>☐ Idiopathic Hypertension<br>☐ Pulmonary Hypertension<br>☐ Congestive heart failure<br>☐ Ischaemic heart disease<br>☐ Congenital heart disease and/or valvular disease<br>☐ Cardiac arrythmia	1.7.2 Obstetric<br>☐ Previous caesarean delivery /myomectomy<br>☐ Pre-eclampsia with severe features or eclampsia<br>☐ PET/ Gestational hypertension<br>☐ Multiple gestation<br>☐ Intrauterine foetal demise<br>☐ Placenta praevia/accreta/abruption<br>☐ Diabetes mellitus on insulin
**1.7.3 Respiratory**<br>☐ Asthma	**1.7.4 Other**<br>☐ Autoimmune disease/Lupus<br>☐ HIV/AIDS<br>☐ Sickle cell disease<br>☐ Bleeding disorder/ coagulopathy/anticoagulation<br>☐ Epilepsy/Cerebrovascular disease/neuromuscular disorder<br>☐ Chronic renal disease<br>☐ Substance use disorder<br>☐ Alcohol abuse
**1.8 Does the patient have a known documented previous history of**	☐ Anxiety<br>☐ Depression<br>☐ PTSD
**1.9 Does the patient have a history of chronic pain?**	☐ Yes<br>☐ No

Part 2 Obstetric Details

**2.1 Gestational age (weeks + days) / 40 Weeks**	**2.2.1 Gravida**	**2.2.2 Parity**
**2.3 Labour category at caesarean delivery**<br>☐ Spontaneous<br>☐ Induced<br>☐ Not in labour	**2.4 Category of caesarean at delivery**<br>☐ Category 1<br>☐ Category 2<br>☐ Category 3<br>☐ Category 4	

Part 3 – Labour analgesia (Complete if applicable, otherwise leave blank. This excludes non-pharmacological methods)	
**3.1.1 Systemic opioids**<br>☐ Pethidine<br>☐ Morphine<br>☐ Diamorphine<br>☐ Other ________________ (please specify)	**3.1.2 Patient controlled analgesia**<br>☐ Remifentanil<br>☐ Other _______________ (please specify)
**3.2.1. Neuraxial analgesia performed**<br>☐ Yes<br>☐ No	**3.2.2. How long ago was the neuraxial inserted**<br>☐ <1 hour<br>☐ 1-4 hours<br>☐ 4-8 hours<br>☐ 8+ hours
**3.3.1 State type of labour neuraxial analgesia**<br>☐ Epidural<br>☐ Combined Spinal Epidural<br>☐ Dural Puncture Epidural<br>☐ Intrathecal catheter<br>**3.3.2 If Combined Spinal Epidural or intrathecal catheter**<br>State drug ___________<br>Dose ____________	**3.4 Labour neuraxial analgesia:**<br>**Please state the grade of inserting clinician:**<br>☐ CT1-3<br>☐ ST4-5<br>☐ ST6-7<br>☐ SAS/Specialty doctor<br>☐ Consultant
**3.5 Drug used for labour epidural analgesia**<br>☐ Low Dose Mix (0.1% Levo + 2mcg/ml Fentanyl)<br>☐ Other __________ **(please specify)**	**3.6 Dosing regime**<br>☐ PCEA (ml bolus and lockout time)<br>☐ PIEB (mls, interval time)<br>☐ CL (ml/hr)<br>☐ Other__________ (please specify)<br>For combined PCEA+PIEB please complete both above

Part 4 – Intraoperative details	
**4.1.1 Time NEURAXIAL insertion or epidural top up (hh:mm)** ________________________<br><br>**4.1.2 Time of Baby DELIVERY (hh:mm)** _________________<br><br>**4.1.3 Time of 'SIGN OUT' e.g. end of surgery (hh:mm)** _________________	
**4.2 Indication for Caesarean (circle all that apply)**<br>☐ Previous caesarean<br>☐ PET<br>☐ Abnormal placentation<br>☐ Abnormal presentation<br>☐ Pelvic/Other MSK indication<br>☐ Maternal request<br>☐ Failure to progress<br>☐ Abnormal CTG<br>☐ Neonatal medical indication<br>☐ Maternal medical indication<br>☐ Other (please state)______________	**4.3 Grade of most senior anaesthetist present during caesarean:**<br>☐ CT1-3<br>☐ ST4-7<br>☐ SAS/Specialty doctor<br>☐ Consultant
**4.4 What was the primary mode of anaesthesia for caesarean?**<br>☐ Epidural top up<br>☐ Spinal<br>☐ CSE<br>☐ DPE<br>☐ Intrathecal catheter<br>☐ Other (please state) ______________________	**4.5 Did you need to convert to a different type of anaesthetic before surgical Incision? (i.e. failure of primary anaesthetic)**<br>☐ Yes<br>☐ No<br>**If yes, please select below**<br>☐ Unplanned epidural/CSE catheter top up<br>☐ Repeat neuraxial<br>☐ Conversion to GA

Part 5 – Medication details. This refers to the medication used for the primary mode of anaesthesia for the caesarean Please give total doses for the procedure.	
**INTRATHECAL drugs/doses:**	
**5.1.1 Local anaesthetic drug/dose/ % conc**<br>☐ Heavy Bupivacaine 0.5%. _______________(ml)	**5..1.2 Opioid drug/dose**<br>☐ Morphine ______________ (mcg)

☐ Plain bupivacaine (ml) 0.5% ________________(ml)<br>☐ Levobupivacaine (ml) 0.5% ________________(ml)<br>☐ Prilocaine (ml) 0.5% ________________(ml)<br>Other (please state) ________________	☐ Fentanyl ________________ (mcg)<br>☐ Diamorphine ________________ (mcg)<br><br>Other (please state) ________________
**EPIDURAL drugs/doses:**	
**5.2.1. Local anaesthetic drug/doses**<br>☐ Bupivacaine (________(%) __________(ml)<br>☐ Levobupivacaine (________(%) __________(ml)<br>☐ Ropivacaine (________(%) __________(ml)<br>☐ Lidocaine (________(%) __________(ml)<br>☐ Other (please state) _______________	**Opioid drug/doses**<br>☐ Fentanyl ________________ (mcg)<br>☐ Morphine ________________ (mg)<br>☐ Diamorphine ________________ (mg)<br>☐ Other (please state)______________

Part 6 – Testing the block

How was the block assessed?

- ☐ See below
- ☐ Not formally assessed
- ☐ Other (please state) ___________________

Document the upper dermatomal level. Please tick the table to indicate which modality was used to check the block. You may have used more than one.

	Left				Right			
Dermatome	Cold	Light touch	Pinprick	Other	Cold	Light touch	Pinprick	Other
T2								
T3								
T4								
T5								
T6								
T7								
T8								
T9								
T10								
T11								
T12								

Other level LEFT ____________ (please state)   Other level RIGHT ____________(please state)

**Motor Block LEFT**<br>**(Please Tick)**	☐ Unable to move feet or knees<br>☐ Able to move feet only<br>☐ Just able to move knees<br>☐ Full flexion of knees and feet
**Motor Block RIGHT**<br>**(Please Tick)**	☐ Unable to move feet or knees<br>☐ Able to move feet only<br>☐ Just able to move knees<br>☐ Full flexion of knees and feet
**Did the patient have intraoperative pain?**	☐ Yes<br>☐ No<br>☐ **If yes, complete Section 7**

Part 7- Intraoperative record and pain

7.1 Please describe any UNPLANNED analgesia, if you had planned to give analgesia via the epidural at the end, for example, this should be documented in P*art* 5.

Time patient reports intraoperative pain (hh:mm)	Stage of procedure. E.g. KTS, delivery gutters, closing	Analgesia / Anxiolytic administered?	If yes, what analgesia/ anxiolytic medications administered in response to this report of pain (please document drugs/ doses)	Resolution of pain	Reassurance given	GA offered?
		Yes/No		Yes/No	Yes/No	Yes/No
		Yes/No		Yes/No	Yes/No	Yes/No
		Yes/No		Yes/No	Yes/No	Yes/No
		Yes/No		Yes/No	Yes/No	Yes/No
		Yes/No		Yes/No	Yes/No	Yes/No

7.2 Indication for supplemental analgesia (tick all that apply):

- ☐ **Inadequate block from start**
- ☐ **Initially adequate block but pain during procedure**
- ☐ **Inadequate duration of block - e.g. duration of surgery**
- ☐ **Failure of communication between patient and anaesthetist, including pre-operative communication**
- ☐ **Other (please specify) ___________________**

7.3.1 Was the primary mode of anaesthesia converted to GA?

- ☐ **Yes**
- ☐ **No**

7.3.2 If yes, at what time was induction (hh:mm) ___________

7.3.3 If yes, what was the indication for conversion to GA (tick all that apply)

- ☐ **Pain**
- ☐ **Surgical requirement – e.g. massive haemorrhage, extended length of procedure**
- ☐ **Anaesthetic complication (e.g. total spinal)**
- ☐ **Other please state _____________**

NIHR | Central London Patient Safety Research Collaboration

UCL

SONAR 1
Snapshot Obstetric National Anaesthetics Research project 1

NHS
University College London Hospitals
NHS Foundation Trust

Snapshot **O**bstetric **N**ational **A**naesthetic **R**esearch Project - **SONAR**

Postoperative caesarean delivery CRF 2 – Day 1 Post delivery. Filled in 24 +/- 6 hours post caesarean delivery. You can fill this in with the patient or ask them to fill Parts 3- themselves and collect afterward.

Script for Investigators
**I am Dr/midwife/research nurse (say name) (if you met earlier for the consent process, please reintroduce yourself. You have consented to be a part of this study and it will take approximately 10 minute to complete.**<br><br>**If the patient agrees please complete the following questions. If the patient cannot talk at this time, please arrange a mutually convenient time to come back, however this needs to be in the data collection timing window for the patient. (24 hours post delivery +/- 6 hours)**

Part 1 – Demographics/study details				
1.1 Study ID ______________<br>(Centre code + ID)				
**1.2 Ethnicity**				
**White**	**Mixed or Multiple ethnic groups**	**Asian, Asian Welsh or Asian British**	**Black, Black British, Black Welsh, Caribbean or African**	**Other**
☐ English, Welsh, Scottish, Northern Irish, British<br>☐ Irish<br>☐ Gypsy or Irish Traveller<br>☐ Roma<br>☐ Other	☐ White & Black Caribbean<br>☐ White & Black African<br>☐ White & Asian<br>☐ Other	☐ Indian<br>☐ Pakistani<br>☐ Bangladeshi<br>☐ Chinese<br>☐ Other	☐ African<br>☐ Caribbean<br>☐ Other	☐ Arab<br>☐ Other ethnic group: Any other ethnic group *please describe*

1.3 Education: what is your highest level of qualification?

- ☐ **No qualifications**
- ☐ **Level 1 and entry level qualifications:** 1 to 4 GCSEs grade A* to C, Any GCSEs at other grades, O levels or CSEs (any grades), 1 AS level, NVQ level 1, Foundation GNVQ, Basic or Essential Skills
- ☐ **Level 2 qualifications:** 5 or more GCSEs (A* to C or 9 to 4), O levels (passes), CSEs (grade 1), School Certification, 1 A level, 2 to 3 AS levels, VCEs, Intermediate or Higher Diploma, Welsh Baccalaureate Intermediate Diploma, NVQ level 2, Intermediate GNVQ, City and Guilds Craft, BTEC First or General Diploma, RSA Diploma
- ☐ **Apprenticeship**
- ☐ **Level 3 qualifications**: 2 or more A levels or VCEs, 4 or more AS levels, Higher School Certificate, Progression or Advanced Diploma, Welsh Baccalaureate Advance Diploma, NVQ level 3; Advanced GNVQ, City and Guilds Advanced Craft, ONC, OND, BTEC National, RSA Advanced Diploma
- ☐ **Level 4 qualifications or above:** degree (BA, BSc), higher degree (MA, PhD, PGCE), NVQ level 4 to 5, HNC, HND, RSA Higher Diploma, BTEC Higher level, professional qualifications (for example, teaching, nursing, accountancy)
- ☐ **Other:** vocational or work-related qualifications, other qualifications achieved in England or Wales, qualifications achieved outside England or Wales (equivalent not stated or unknown)

1.4 Employment:

- ☐ Employee
- ☐ Self employed with employees
- ☐ Self employed without employees
- ☐ Unemployed, seeking work or waiting to start
- ☐ Student
- ☐ Looking after home or family
- ☐ Long-term sick or disabled

Is your work full or part time?

- ☐ Full time
- ☐ Part time
- ☐ Not applicable

1.5 Health: How would you describe your general health?

- ☐ Very good
- ☐ Good
- ☐ Fair
- ☐ Bad
- ☐ Very bad

1.6 Housing: How would describe your current housing situation?

- ☐ Own your home outright
- ☐ Own it with help of a mortgage or loan
- ☐ Pay part rent and part mortgage (shared ownership)
- ☐ Rent
- ☐ Live there rent free (including rent free in a relative or friend's property (excluding squatting)
- ☐ Prefer not to say

1.7 Disability: Would you describe yourself as disabled?

- ☐ Yes
- ☐ No

Part 2 – Intraoperative pain

2.1.1 Did you have pain during the operation to delivery your baby?

- ☐ Yes
- ☐ No

2.1.2 If yes, when your pain was at its worst, how bad was it?
0 = no pain, 10 = worst pain imaginable

0	1	2	3	4	5	6	7	8	9	10

2.1.3 From these options, when did your pain occur:

- ☐ Right at the beginning of surgery/ First incision
- ☐ Before your baby was delivered
- ☐ While your baby was being delivered
- ☐ After your baby was delivered

If after your baby was delivered, further clarify:

- ☐ Soon after your baby was delivered
- ☐ Close to the end of surgery
- ☐ Unsure

2.1.4 Please circle any number that corresponds to where on the body they felt the pain

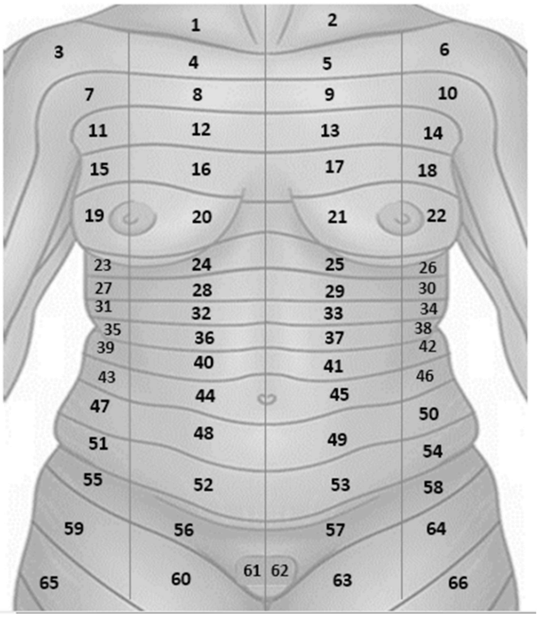

Part 3 – Post Delivery pain scores

NIHR | Central London Patient Safety Research Collaboration

UCL

SONAR 1
Snapshot Obstetric National Anaesthetics Research project 1

NHS
University College London Hospitals
NHS Foundation Trust

3.1 What is the severity of pain you have experienced in the last 24 hours (+/- 6 hours) (verbal rating score 0 – 10)
0 = no pain, 10 = worst pain imaginable

At rest	0	1	2	3	4	5	6	7	8	9	10
On moving	0	1	2	3	4	5	6	7	8	9	10

3.2 How would you rate your general health state in the last 24 hours?

Worse imaginable health state — **Best imaginable health state**

Please mark with a cross on the scale above how you have been feeling in the last 24 hours

For investigator to complete:

Global health VAS score ____/100

Part 4 Maternal Satisfaction Scale

This next part asks you to reflect on your experience in the operating theatre, not how you feel now

No.	Response							
**ANAESTHETIC:**		**Strongly disagree**						**Strongly agree**
**1**	I was pain free in the operating room	1	2	3	4	5	6	7
**2**	I felt the anaesthetic I received was safe for me	1	2	3	4	5	6	7
**3**	I felt the anaesthetic I received was safe for my baby.	1	2	3	4	5	6	7
**4**	I had no pain when the needle was put into my back.	1	2	3	4	5	6	7
**INSERTION OF NEEDLE IN BACK:**								
**5**	The needle was put easily into my back.	1	2	3	4	5	6	7
**6**	I was in a comfortable position when the needle was put into my back.	1	2	3	4	5	6	7
**SIDE EFFECTS: In the operating room I did NOT experience the following:**		**Strongly disagree**						**Strongly agree**
**7**	Shivering	1	2	3	4	5	6	7
**8**	Dry lips/mouth	1	2	3	4	5	6	7
**9**	Dry throat	1	2	3	4	5	6	7
**10**	A change in mood	1	2	3	4	5	6	7
**At 24 hours (+/- 6 hours) after childbirth, I have NOT experienced:**								
**11**	Back problems	1	2	3	4	5	6	7
**12**	Itchiness	1	2	3	4	5	6	7
**ATMOSPHERE: In the operating room, during the surgery, I was able to:**		**Strongly disagree**						**Strongly agree**
**13**	Interact with my partner	1	2	3	4	5	6	7
**14**	Bond with the baby	1	2	3	4	5	6	7
**15**	Have a sense of control.	1	2	3	4	5	6	7
**16**	Communicate with the staff	1	2	3	4	5	6	7
**17**	See the baby after delivery	1	2	3	4	5	6	7

**18**	Hold the baby after delivery	1	2	3	4	5	6	7
**19**	I knew what the staff were doing during the operation/childbirth	1	2	3	4	5	6	7
**20**	I found the atmosphere in the operating room comfortable.	1	2	3	4	5	6	7
**21**	I was able to feed my baby after delivery.	1	2	3	4	5	6	7
**22**	I recovered quickly after caesarean.	1	2	3	4	5	6	7
**FOR INVESTIGATOR TO COMPLETE:<br>MATERNAL SATISFACTION SCORE /154**								

Part 5 – Maternal Comfort

This next part asked to reflect on your experience in the operating theatre, <u>not</u> how you feel now

**No**	Response	**Strongly disagree**					**Strongly agree**	
**1**	I had a positive delivery experience in the operating room	1	2	3	4	5	6	7
**2**	I could understand everything the anaesthetist told me during surgery	1	2	3	4	5	6	7
**3**	I received sufficient support from the staff in the operating room	1	2	3	4	5	6	7
**4**	I had no pain during my caesarean delivery in the operating room.	1	2	3	4	5	6	7
**5**	I got enough pain relief during my caesarean section in the operating room	1	2	3	4	5	6	7

Part 6 Assessment of anxiety. Think of the time between the decision to have a caesarean and the operation itself.

I would give my anxiety before my caesarean delivery a score of:

0 1 2 3 4 5 6 7 8 9 10

Part 7 Communication rating/feedback

If you had pain how would assess the communication of options for pain relief during surgery?

Numerical reporting scale where:
0 = worst imaginable communication
100 = best possible communication

	**Yes**	**No**	**Not sure**
**Did you tell your anaesthetist that you had pain in the operating room?**			
**Were you offered more pain relief in the operating room?**			

Is there anything else you would like the research team to know? Please feel free to add any additional concerns or text here:

Snapshot Obstetric National Anaesthetic Research Project - **SONAR**

Postoperative caesarean delivery CRF 3 – Medium term outcomes.
Completed 6 weeks +/- 3 days post caesarean delivery via telephone call with patient. Thank you for your time and effort.

Script for Investigators

I am Dr/midwife/research nurse (say name) (if you met earlier for the consent process, please reintroduce yourself.

You have consented to be a part of this study and it will take approximately 10-15 mins to complete. If the patient agrees please complete the following questions. If the patient cannot talk at this time, please arrange a mutually convenient time to come back, however this needs to be in the data collection timing window for the patient. (6 weeks hours post delivery +/- 3 days)

Part 1 Study details

**Study ID**	~~Date time CRF 3 completion (dd/mm/yyyy) (hh:mm)~~

Part 2 Postpartum pain

What is the severity of the pain you have experienced the last 24 hours (at 6 weeks post-delivery +/- 3 days)?
0 = no pain, 10 = worst pain imaginable

At rest	**0**	**1**	**2**	**3**	**4**	**5**	**6**	**7**	**8**	**9**	**10**
On moving	**0**	**1**	**2**	**3**	**4**	**5**	**6**	**7**	**8**	**9**	**10**

Part 3 Edinburgh Postnatal Depression scale

Notes to Investigators:

Postpartum depression is the most common complication of childbearing. The 10-question Edinburgh Postnatal Depression Scale (EPDS) is a valuable and efficient way of identifying patients at risk for "perinatal" depression.
EPDS score should not override clinical judgment.

If investigators find a score of more than 11, or answers ANYTHING other than 'never'; to question 10:
Please encourage your patient to make a GP appointment to discuss this further. Please inform them that we will be sending them a letter with more resources and also their GP a letter informing them of the score.

Instructions for using the Edinburgh Postnatal Depression Scale:

1. **The mother is asked to give the response that comes closest to how they have been feeling in the previous seven days;**
2. **All the items should be completed;**
3. **Care should be taken to avoid the possibility of the respondent discussing their answer with others. Answers should come from the participant themselves, unless they have limited English or difficulty with comprehension.**

Those with postpartum depression need not feel alone. They may find useful information on the websites of the Pre and Postnatal Depression Advice and Support (PANDAS) and from groups such as Maternal Mental Health Alliance website and https://www.birthtraumaassociation.org.uk.

Each question is given a score of 0-3. Answer 'a' is 0 and answer 'd' is a 3. The maximum score is 30.

SCRIPT FOR INVESTIGATOR
As you have recently had a baby in approximately the last 6 weeks, we would like to know how you are feeling. Please interrupt me if you do not understand something or if things are not clear to you. There are no right or wrong answers. Please give the answer that comes close to how you have felt IN THE PAST SEVEN DAYS, not just how you have felt since your caesarean section, or how you feel today. First, I am going to read out ten questions. Each question has a choice of four answers. Do not choose more than one answer.

Note to interviewer: first read all four options for each question. Then ask the respondent to choose which one applies to themselves. Repeat the question and options if necessary. Mark the appropriate box under each heading. You may need to remind the respondent regularly that the timeframe is THE PAST SEVEN DAYS

**1**	In the past seven days:	a. As much as I always could

	I have been able to laugh and see the funny side of things	b. Not quite so much now<br>c. Definitely not so much now<br>d. Not at all
**2**	I have looked forward with enjoyment to things	a. As much as I ever did<br>b. Rather less than I used to<br>c. Definitely less than I used to<br>d. Hardly at all
**3**	I have blamed myself unnecessarily when things went wrong	a. Yes, most of the time<br>b. Yes, some of the time<br>c. Not very often<br>d. No, never
**4**	I have been anxious or worried for no good reason	a. No, not at all<br>b. Hardly ever<br>c. Yes, sometimes<br>d. Yes, very often
**5**	I have felt scared or panicky for no very good reason	a. Yes, quite a lot<br>b. Yes, sometimes<br>c. No, not much<br>d. No, not at all
**6**	Things have been getting on top of me	a. Yes, not of the time I have been able to cope at all<br>b. Yes, sometimes I haven't been coping as well as usual<br>c. No, most of the time I have coped quite well<br>d. No, have been coping as well as ever
**7**	I have been so unhappy that I have had difficulty sleeping	a. Yes, most of the time<br>b. Yes, sometimes<br>c. Not very often<br>d. No, not at all
**8**	I have felt sad or miserable	a. Yes, most of the time<br>b. Yes, quite often<br>c. Not very often<br>d. No, not at all
**9**	I have been so unhappy that I have been crying	a. Yes, most of the time<br>b. Yes, quite often<br>c. Only occasionally<br>d. No, never
**10**	The thought of harming myself has occurred to me	a. Yes, quite often<br>b. Sometimes<br>c. Hardly ever<br>d. Never
**For the investigator to complete EPDS score _____/ 30**		

Part 4 – GAD7 – The Generalised Anxiety Disorder Assessment

Notes to Investigators:

The GAD-7 is a valid and efficient tool for screening for Generalised Anxiety Disorder. Patients who score above 15 are likely to be suffering from a severe anxiety disorder. The GAD7 score should not override clinical judgment.

If investigators find a score of more than 15:
Please encourage your patient to make a GP appointment to discuss this further. Please inform them that we will be sending them a letter with more resources and also their GP a letter informing them of the score.

Instructions for using the GAD7:

The patient is asked to give the response that comes closest to how they have been feeling in the previous TWO WEEKS

Scoring GAD-7 Anxiety severity

This is calculated by assigning scores of 0, 1, 2, and 3 to the response categories, respectively, of "not at all," "several days," "more than half the days," and "nearly every day." GAD-7 total score for the seven items ranges from 0 to 21.

**0–4: minimal anxiety, 5–9 mild anxiety; 10–14 moderate anxiety; 15–21: severe anxiety**
**SCRIPT FOR INVESTIGATORS:**<br><br>**Next, I am going to read out seven questions. Each question has a choice of four answers. Do not choose more than one answer. I can repeat the question and let me know if anything is not clear. There are no right or wrong answers. Please give the answer that comes close to how you have felt IN THE PAST TWO WEEKS, not just how you have felt since your caesarean section, or how you feel today.**<br><br>**(Note to interviewer: first read all four options for each question. Then ask the respondent to choose which one applies to themselves. Repeat the question and options if necessary. Mark the appropriate box under each heading. You may need to remind the respondent regularly that the timeframe is THE PAST TWO WEEKS)**

**Over the last two weeks, how often have you been bothered by the following problems?**	**Not at all**	**Several Days**	**More than half the days**	**Nearly every day**
**1) Feeling nervous, anxious, or on the edge**	0	1	2	3
**2) Not being able to stop or control worrying**	0	1	2	3
**3) Worrying too much about different things**	0	1	2	3
**4) Trouble relaxing**	0	1	2	3
**5) Being so restless that its hard to sit still**	0	1	2	3
**6) Becoming easily annoyed or irritable**	0	1	2	3
**7) Feeling afraid, as if something awful might happen**	0	1	2	3
**For investigators to complete: GAD7 score ____/ 21**				

Part 4 PCL-5 Post Traumatic Stress Disorder Checklist

Notes for investigators:
PCL-5 is a validated tool to screen for Post traumatic stress disorder. Mothers who score above 30 are likely to be suffering from a severe anxiety disorder. The PCL- score should not override clinical judgment.

If investigators find a score of more than 30:
Please encourage your patient to make a GP appointment to discuss this further. Please inform them that we will be sending them a letter with more resources and also their GP a letter informing them of the score.

Instructions for using the PCL-5: The patient is asked to give the response that comes closest to how they have been feeling in the previous ONE MONTH

SCRIPT FOR INVESTIGATORS:

Next is a list of twenty problems that people sometimes have in response to a very stressful experience. For the purposes of this questionnaire the stressful experience relates to your experiences in the operating room during childbirth. Each question has a choice of five answers. Do not choose more than one answer. I can repeat and let me know if anything is not clear. There are no right or wrong answers. Please give the answer that comes close to how you have felt IN THE PAST ONE MONTH not just how you have felt since your caesarean section, or how you feel today.

(Note to interviewer: first read all five options for each question. Then ask the respondent to choose which one applies to themselves. Repeat the question and options if necessary. Mark the appropriate box under each heading. You may need to remind the respondent regularly that the timeframe is THE PAST MONTH)

Q	Response	Not at all	A little bit	Moderately	Quite a bit	Extremely
1	Repeated, disturbing, and unwanted memories of the stressful experience?	0	1	2	3	4
2	Repeated, disturbing dreams of the stressful experience?	0	1	2	3	4
3	Suddenly feeling or acting as if the stressful experience were actually happening again (as if	0	1	2	3	4

	you were actually back there reliving it)?					
4	Feeling very upset when something reminded you of the stressful experience?	0	1	2	3	4
5	Having strong physical reactions when something reminded you of the stressful experience (for example, heart pounding, trouble breathing, sweating)?	0	1	2	3	4
6	Avoiding memories, thoughts, or feelings related to the stressful experience?	0	1	2	3	4
7	Avoiding external reminders of the stressful experience (for example, people, places, conversations, activities, objects, or situations)?	0	1	2	3	4
8	Trouble remembering important parts of the stressful experience?	0	1	2	3	4
9	Having strong negative beliefs about yourself, other people, or the world (for example, having thoughts such as: I am bad, there is something seriously wrong with me, no one can be trusted, the world is completely dangerous)?	0	1	2	3	4
10	Blaming yourself or someone else for the stressful experience or what happened after it?	0	1	2	3	4
11	Having strong negative feelings such as fear, horror, anger, guilt, or shame?	0	1	2	3	4
12	Loss of interest in activities that you used to enjoy?	0	1	2	3	4
13	Feeling distant or cut off from other people?	0	1	2	3	4
14	Trouble experiencing positive feelings (eg, being able to feel happiness or have loving feelings for people close to you?)	0	1	2	3	4
15	Irritable behavior, angry outbursts, or acting aggressively?	0	1	2	3	4
16	Taking too many risks, or doing things that could cause you harm?	0	1	2	3	4
17	Being ‘super-alert’ or watchful or on guard.	0	1	2	3	4
18	Feeling jumpy or easily startled?	0	1	2	3	4
19	Having difficulty concentrating?	0	1	2	3	4
20	Trouble falling or staying asleep?	0	1	2	3	4
**For investigator to complete: PCL –5 score _______/80**						
