# Supplementary material for: Snapshot of Obstetric National Audit and Research Project (SONAR1): aprotocol for an international observational cohort study
Source: BMJ Open. 2025 Jun 24;15(6):e103525. doi: 10.1136/bmjopen-2025-103525 (PMC12198837; doi:10.1136/bmjopen-2025-103525)
Supplement: Supplementary file 3 [file bmjopen-2025-103525.reviewer_comments.pdf]

PEER REVIEW HISTORY

BMJ Open publishes all reviews undertaken for accepted manuscripts. Reviewers are asked to complete a checklist review form and are provided with free text boxes to elaborate on their assessment. These free text comments are reproduced below.

ARTICLE DETAILS

Title (Provisional)

SONAR1: Snapshot of Obstetric National Audit and Research Project – Protocol for an International Observational Cohort Study

Authors

Patel, Reshma; O'Carroll, James; Kua, Justin; Sultan, P; Carvalho, Brendan; El Sharawi, Nadir; Eley, Victoria; O'Brien, Pat; Stanford, Susanna; Hill, Samantha; Craig, Robert; Lucas, D N; Hou, Bo; Moonesinghe, S. Ramani

---

This article was not externally reviewed at BMJ Open. Protocols that have been independently assessed prior to submission to BMJ Open are usually fast-tracked to publication on the grounds that further substantial changes will not be possible. This independent assessment will usually be external, independent review for both a substantial grant award from a non-commercial or government funder and ethics approval.
